# Time to review reflective practice?

**DOI:** 10.1093/intqhc/mzac052

**Published:** 2022-05-27

**Authors:** Terry Quilty, Lyn Murphy

**Affiliations:** Manukau Institute of Technology, Private Bag 94006 Manukau, Auckland 2241, New Zealand; Health Sciences, Auckland University of Technology (AUT), Private Bag 92006, Auckland 1142, New Zealand

**Keywords:** reflective practice, learning, education, perception

## Abstract

**Background:**

Reflective practice is an integral part of modern healthcare. If done well, it can significantly improve the individual skills of health care practitioners. However, we hypothesize that extrapolating individual reflective practice into broader organization applications undermines its fundamental nature and inhibits objective benchmarking within the health sector.

**Methods:**

We reflect on the nature and use of the reflective practice in healthcare.

**Results:**

An organization that practices reflective practice may, in effect, create an environment where reflective practice is promoted but operates to homogenize thinking to a point where it turns into dysfunctional institutional navel-gazing. Homogenized thinking may inhibit the ability to move beyond practice to explore ideas that lead to change.

**Conclusions:**

The collective approach to reflective practice can subvert the underlying process of self-analysis, which allows the critical examination of individual values, priorities, and evaluations. It can inhibit individual growth, favouring a homogenizing effect which is the antithesis of an innovative organization when measured against the original intent and must therefore be used with care.

The development of reflective practices traces back to Dewey [1], where the individual applied a subjective assessment (the reflection), measured against objective facts. We adopt Boyd and Fales’s [2] definition of a process of internally examining and exploring an issue of concern, triggered by an experience, which creates and clarifies meaning in terms of self and results in a changed conceptual perspective. The development of many approaches is well documented [3]. A 2015 review [4] looked at the models then in practice, identifying that ‘reflection appears to be influenced more by a reductionist approach aligned with dominant epistemological positions in medicine, such as evidence-based medicine, than by the historically critical (artistic) philosophical underpinnings’. We would also suggest a dichotomy exists in the application of reflective practice as a training tool in evidence-based medicine and the application as a learning tool. One is training, and one is reflection. This is exacerbated by the application of group reflective practice. The question becomes whether training and reflective practice may become confused or mixed or substituted to the detriment of both.

McVey and Jones [5] studied a clinical supervision group where staff reflected on their practice in a non-judgemental environment. The participants’ positive reports are put forward to support reflective practice. We question whether it may instead support a group reflection as an element of clinical training. A risk here is that where expert facilitation is not available, regular or consistent, the practice may confuse training with reflection. Reflection is not a substitute for training, and the additional question becomes how those reflecting can be aware of the best practice if the benchmark used is the group practice.

Rhetoric, definition and interpretation may be a central issue if reflective practice becomes more entrenched in the training schema and accepted for regulatory compliance, which can be explicit or implicit depending on the regulation.

Group reflective practice is also mandated in policy statements. For example, the National Health Service (NHS) Scotland policy for facilitated group reflective practice promotes regular inter-disciplinary group reflection [6]. This policy does make a clear distinction between clinical supervision and facilitated reflective practice, recognizing explicitly that substitution should not occur. The Scottish NHS has a clinical focus and may explicitly exclude reflective learning, an example of the reductionist approach Fagkros identifies. In contrast, the Nurses and Midwifery council require reflective learning for revalidation applicants evidenced by five examples of how this has been achieved [7]. These positions are not readily reconcilable. Arguably, the applicants could use the group reflective practice to illustrate their own learning; however, this begs the question of whether group reflective practice is an individual reflection. The individual has ‘learnt from’, not learnt reflectively and has simply adopted the group approach.

Where this argument becomes important is in the regulatory compliance questions. Regulatory requirements are there for safety and performance. Where a mandatory requirement may be met by a practice that has no specified definition, the potential for differing approaches may result in inconsistent applications, all of which are ostensibly meeting the mandated requirement. In this regard, we observe a process designed for a unique application being redesigned and applied to a group further, compounded by being either with or without expert facilitation. They may not meet the regulatory aim, which for the most part will target the evidence-based medicine outcomes. In addition, if resources are limited and the group facilitation is not independent or expert or industry informed, the perspective is entirely internal. It is navel gazing.

The second perspective raised is whether group or organizational reflection is of value as a reflective practice to the individual or whether group or organizational reflective practice is undermining the reflection and learning. In this context, the reference to organizational practice is to group reflective practice, where the organisation policy defines, directs or restricts the reflection as exemplified in the Scotland NHS Group Reflective Practice policy. In a group, a consensus will form explicitly or implicitly and the group think element will inevitably intrude particularly for the newer practitioners. It is in this sense we assert there is an organizational reflective practice to be differentiated from individual reflection. We do not refer to the organization entity itself reflecting, rather that the organization practice mechanisms create a collective view (or group views), which then represents the organization and becomes its benchmarks.

Reflective practice is an integral part of modern healthcare. If done well, it can significantly improve the individual skills of healthcare practitioners [8]. However, we hypothesize that health professionals who engage in group reflective practice may inadvertently (or intentionally) homogenize thinking, internalize benchmarking and place the organization at risk of fostering institutional navel gazing. Another way to pose this question is to ask whether the extrapolation of individual reflective practice into broader organization applications undermines its fundamental nature and objective benchmarking within the health sector.

The extrapolation of this thinking is an organizational approach as distinguished from the individual as the focal point. The organizational approach ceases to be one of the individual collective effects that improve individual performance. Instead, it morphs into a collective self-reflection, demonstrated in the methods used and how they focus not on individual practice but teams, structures and systems.

Extending reflective practice to groups initially appears to be a logical extrapolation but may effectively be a subversion of the individuals’ reflective practice whereby the collective and organizational benefit was a derivative. This is replaced by one where the organization’s collective self-reflection drives and contains both the individual and organizational subjective reflection (the institutional position).

Reviewing an experience does not always lead to effective reflection [9]. Reflective practice for the individual is both a subjective and an objective approach to improvement. The inward measurement is bounded by external consideration of benchmarks and best practice and, as such, is not ‘navel gazing’. However, applied at an organizational level, it may negate such objectivity in favour of an isolationist approach that excludes industry best practices and consideration of external inputs or considerations (for example, exploring policy issues from the perspectives of indigenous peoples and adopting digital technologies or different modes of delivery such as hospital at home or interprofessional practice). In this way, the collective approach to reflective practice may subvert self-analysis and inhibit individual growth in favour of homogenization.

## Conclusion

Reflective practice is valuable. Where group reflective practice is used for group reflection, resourced and facilitated expertly and related to external benchmarks, the potential benefits are extensive. The various definitions and approaches confound the issues by allowing for both divergent and contradictory approaches. These interpretations allow for both expansive and reductionist application. This lack of clarity and the breadth of potential approaches import a risk where reflective practice is recognized as a mechanism for meeting clinical, operational or regulatory needs. This risk is magnified where resourcing may not allow the level of rigour indicated by that need. In a resource-constrained world, mandated group reflective practice is an expeditious option and one which is both hard to measure and critique. Care, clarity of definition and application, and resourcing are needed. If it is not possible to have that rigour in group reflective practice, then we suggest that a more rigorous specified and measureable mechanism may be appropriate. The individual need is the growth of that person and promoting innovative thought and awareness. This is not necessarily the need of the group. The group approach, homogenising reflection as a single group view, negates individual growth and divergent thinking. In this environment innovation that would otherwise occur is subsumed. This is a negative impact viewed from an innovative perspective, but may be a desirable aim where, such as in clinical situations, consistent thought and action is the primary aim. This homogenization is, from a practice perspective, arguably an aim in clinical supervision, whereas in education, critical thinking and problem solving are important. Resources and expediency may necessitate group practice but cannot be assumed to be individually reflective and the group reflection, while valuable organizationally and individually, may not be transferable to the individual reflective practice required.

## References

[1] Dewey J . *How We Think: A Restatement of the Relation of Reflective Thinking to the Educative Process*. Boston, MA: D.C. Heath & Co Publishers, 1933.

[2] Boyd EM, Fales AW. Reflective learning: key to learning from experience. *J Humanist Psychol* 1983;23:99–117.

[3] Sellars SM . [*Internet*]. Sage, 2017. Available from: https://uk.sagepub.com/sites/default/files/upm-binaries/59229_Sellars.pdf.

[4] Fragkos KC . Reflective practice in healthcare education: an umbrella review. *Educ Sci* 2016;6:27.

[5] McVey J, Jones T. Assessing the value of facilitated reflective practice groups. *Cancer Nurs Pract (Through 2013)* 2012;11:32–7.

[6] The State Hospitals Board for Scotland. Reflective Practice Groups for the MDT Policy. NHS Scotland. https://www.tsh.scot.nhs.uk/Policies/Docs/CP52%20-%20Reflective%20Practice%20Groups%20for%20the%20MDT%20Policy.pdf (6 June 2022, date last accessed).

[7] The Nursing and Midwifery Council. Guidance Sheet Reflective Practice. https://www.nmc.org.uk/globalassets/sitedocuments/revalidation/reflective-practice-guidance.pdf (6 June 2022, date last accessed).

[8] Koshy K, Limb C, Gundogan B et al. Reflective practice in health care and how to reflect effectively. *Int J Surg Oncol (N Y)* 2017;2:e20. 10.1097/IJ9.0000000000000020 PMC5673148 29177215

[9] Figueroa CA, Harrison R, Chauhan A et al. Priorities and challenges for health leadership and workforce management globally: a rapid review. *BMC Health Serv Res* 2019;19:239. 10.1186/s12913-019-4080-7 PMC6480808 31014349

